# Iodine-125 seeds combined with anlotinib in the treatment of recurrent retroperitoneal liposarcoma after surgery: a case report

**DOI:** 10.3389/fonc.2025.1540868

**Published:** 2025-04-28

**Authors:** Gaoyan Tang, Yuping Zhang, Wenjuan Meng, Shoubin Zhong, Hui Feng, Guohua Yu, Shuzhen Liu, Rui Li

**Affiliations:** ^1^ Department of Oncology, Weifang People’s Hospital (The First Affiliated Hospital of Shandong Second Medical University), Weifang, China; ^2^ Center for Precision Pathological Diagnosis, Weifang People’s Hospital (The First Affiliated Hospital of Shandong Second Medical University), Weifang, China

**Keywords:** liposarcoma, surgery, recurrence, iodine-125 seeds, anlotinib

## Abstract

Retroperitoneal liposarcoma (RPLS) is a rare malignant mesenchymal tumor originating in the retroperitoneal space. It is characterized by a low incidence, poorly understood etiology and pathogenesis, and diverse imaging and pathological manifestations. The malignancy of RPLS varies significantly among cases. Currently, surgical resection remains the primary treatment for primary retroperitoneal liposarcoma; however, the disease is associated with a high and rapid recurrence rate, which severely impacts patient prognosis. This study presents a case of recurrent retroperitoneal liposarcoma treated with iodine-125 seed implantation following surgical intervention. Due to the large tumor size, high risk of postoperative recurrence, and the challenges of accurately targeting postoperative radiotherapy, surgical re-intervention was deemed unsuitable. Consequently, a comprehensive treatment plan involving iodine-125 seed implantation combined with anlotinib therapy was formulated. The patient achieved stable disease control over a 3-year follow-up period, demonstrating the potential efficacy of this combined therapeutic approach. This case highlights the antitumor potential of iodine-125 seed implantation combined with anlotinib in the management of retroperitoneal liposarcoma, particularly in cases where surgical options are limited.

## Introduction

Soft tissue sarcoma (STS) is a rare malignancy. Among all STS cases, liposarcoma accounts for approximately 20%. Liposarcoma, originating from mesenchymal tissue, predominantly distributes in the extremities and retroperitoneal region (1). Retroperitoneal liposarcoma specifically emanates from the adipose tissue within the retroperitoneal space, rather than from a particular organ (2). It represents around 54.2% of all liposarcoma cases. The incidence rate is approximately 2.5 cases per million population, with a slightly higher prevalence in males compared to females (3). Due to its profound anatomical location, insidious onset, and absence of conspicuous early - stage manifestations, it is typically diagnosed only when the tumor has grown to a size sufficient to invade or compress adjacent tissues and organs, such as the renal capsule and the aorta. This scenario often culminates in a significantly more unfavorable prognosis (4).

Iodine - 125 seed implantation is a distinct modality within the realm of brachytherapy. During the treatment procedure, radioactive iodine - 125 seeds are meticulously implanted either directly into the tumor parenchyma or in its immediate vicinity (5). Generally, brachytherapy is defined by the placement of a radiation source in close proximity to the target tissue. This strategic positioning enables the efficient delivery of a high - intensity radiation dose to the tumor while simultaneously aiming to minimize the radiation exposure of the surrounding healthy tissues. Brachytherapy has proven its efficacy across a wide array of cancer types and currently, combination therapies integrating iodine - 125 seed implantation with chemotherapy, targeted therapy, immunotherapy, and thermotherapy have demonstrated promising clinical outcomes and enhanced overall survival rates (6–8).

The present study details a case of recurrent retroperitoneal liposarcoma following surgery. The tumor recurred shortly after the initial operation, rendering complete re - resection challenging. Additionally, the patient had poor tolerance. As a result, second - stage surgery was not considered. Consequently, CT - guided implantation of iodine - 125 seeds into metastatic lesions was regarded as the most viable treatment option. Subsequently, the treatment goal of maintaining stable disease over a three - year period was successfully achieved.

## Case description

**Figure 1:**
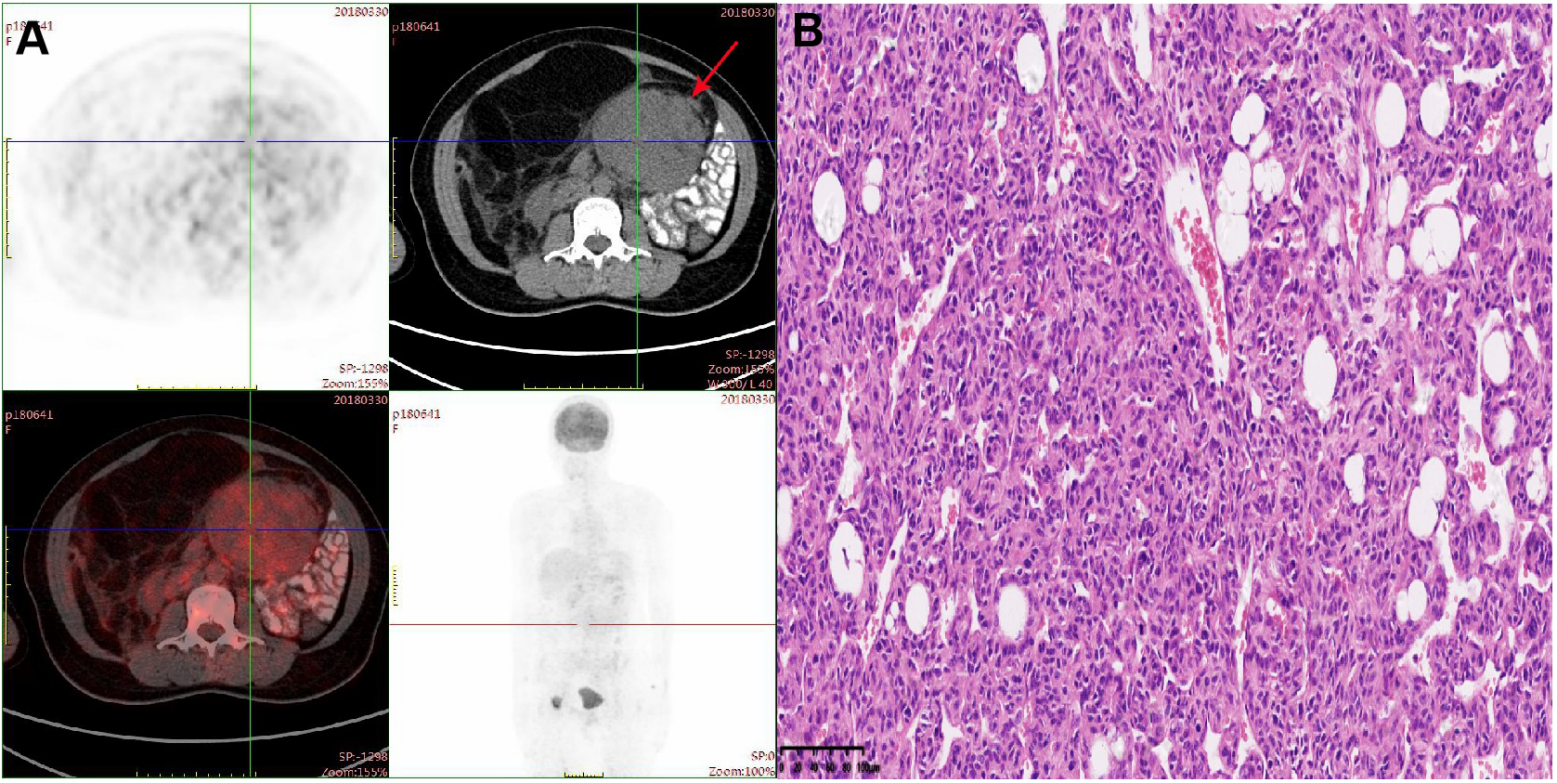
**(A)** PET-CT scan demonstrated a mass with peripheral elevated FDG avidity in the left lower abdomen. **(B)** Hematoxylin-eosin (HE) staining of postoperative tumor tissue (20×).

A 37-year-old woman with a history of tension-free inguinal hernia patch repair and retroperitoneal lipoma resection was referred to our hospital in March 2018 due to a large abdominal mass measuring approximately 15 cm, accompanied by abdominal fullness and discomfort. PET-CT revealed a massive lesion in the left lower abdomen without distant metastasis or infiltration into adjacent critical organs (**Figure 1A**). Six days later, the patient underwent surgical resection under general anesthesia. Intraoperatively, a large tumor measuring approximately 20 × 20 cm was identified in the pelvic abdominal wall and abdominal cavity. The tumor was densely adherent to the right ovary and fallopian tube, but no metastatic nodules were observed in the liver or pelvic region, and no enlarged lymph nodes were detected. The patient underwent abdominal-pelvic tumor resection combined with right salpingo-oophorectomy. Postoperative pathological examination revealed a mesenchymal tumor with atypical cells and approximately 5 mitotic figures per 10 high-power fields (HPF) (**Figure 1B**). Immunohistochemical analysis yielded the following results: Vimentin (+), Bcl-2 (+), S-100 (partial+), CD68 (partial+), CK (-), α-inhibin (-), CD117 (-), Dog-1 (-), CD34 (vascular+), and Ki-67 (10%). Based on the pathological and immunohistochemical findings, the final diagnosis was well-differentiated liposarcoma.

**Figure 2:**
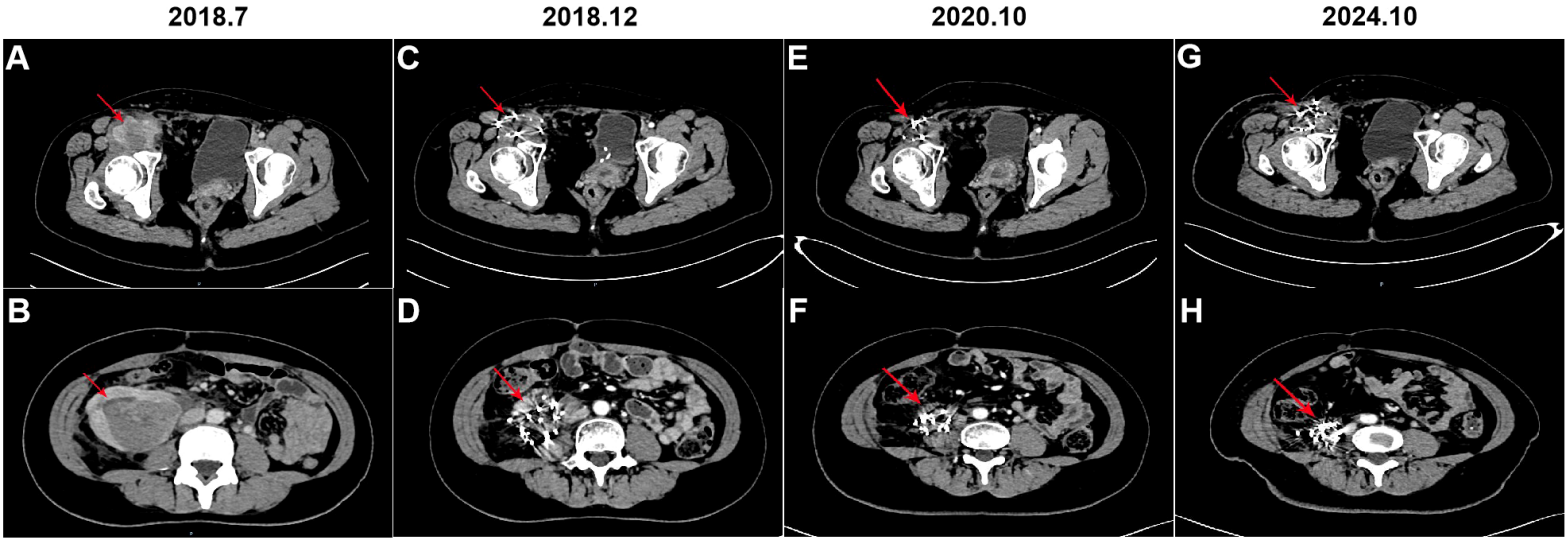
Computerized tomography (CT) was performed before and after iodine-125 seeds implantation therapy. **(A, B)** CT scan of the mass in the right groin and right abdomen, respectively (arrow); **(C, D)** CT scan of the mass in the right groin and right abdomen after 5 months of iodine-125 seeds implantation, respectively (arrow); **(E, F)** CT scan of the mass in the right groin and right abdomen after 3 years of iodine-125 seeds implantation, respectively (arrow); **(G, H)** CT scan of the mass in the right groin and right abdomen after 6 years of iodine-125 seeds implantation, respectively (arrow).

Forty days postoperatively, the patient developed needle-like pain in the right groin, which was radiating to the anterior right thigh and hip. CT scans demonstrated irregular soft tissue and fat density masses in the right groin and abdomen, with the largest mass in the right mid-abdomen measuring approximately 7.7 cm in diameter (**Figures 2A, B**). In the event of liposarcoma recurrence, re - surgical intervention may be contemplated. When assessing the patient’s current physical status, retroperitoneal liposarcoma is typically in close proximity to vital intra - abdominal organs or structures. Surgical procedures often face challenges in achieving wide - margin resection, thereby conferring a high probability of recurrence. In light of a comprehensive evaluation of the patient’s condition, the multidisciplinary team (MDT) recommends iodine - 125 brachytherapy seed implantation for treatment. Consequently, CT-guided iodine-125 seed implantation for the right groin and retroperitoneal liposarcoma was performed in July 2018. For the right groin mass, which measured approximately 4.9 cm × 4.6 cm × 2.7 cm, a total of 187 iodine - 125 seeds, each with an activity of 0.7 mCi, were implanted in accordance with a prescribed radiation dosage of 120 Gy. In the case of the right retroperitoneal mass, with dimensions of approximately 6.1 cm × 8.2 cm × 8.6 cm, 195 iodine - 125 seeds having an activity of 0.5 mCi each were implanted, adhering to a radiation prescription of 120 Gy. To further effectively prevent tumor recurrence, the patient commenced targeted therapy with anlotinib. The initial dosing regimen was set at 12 mg per day. After 14 consecutive days of administration, a 7 - day drug - free interval was implemented, thus constituting a complete 21 - day treatment cycle. Throughout the treatment period, routine hematological tests, liver and kidney function tests, five - parameter thyroid function tests, and routine urinalysis were regularly and strictly carried out in accordance with established protocols. Blood pressure was also monitored regularly. During this process, the patient was diagnosed with asymptomatic Grade 1 hypothyroidism. Six months into the treatment, considering the patient’s development of Grade 2 hand - foot syndrome, after careful evaluation, the single - dose was adjusted to 10 mg per day. Upon subsequent observation, the patient demonstrated good tolerance to the adjusted dosage and the patient received anlotinib treatment for a total of 3 years until the disease progressed.

**Figure 3:**
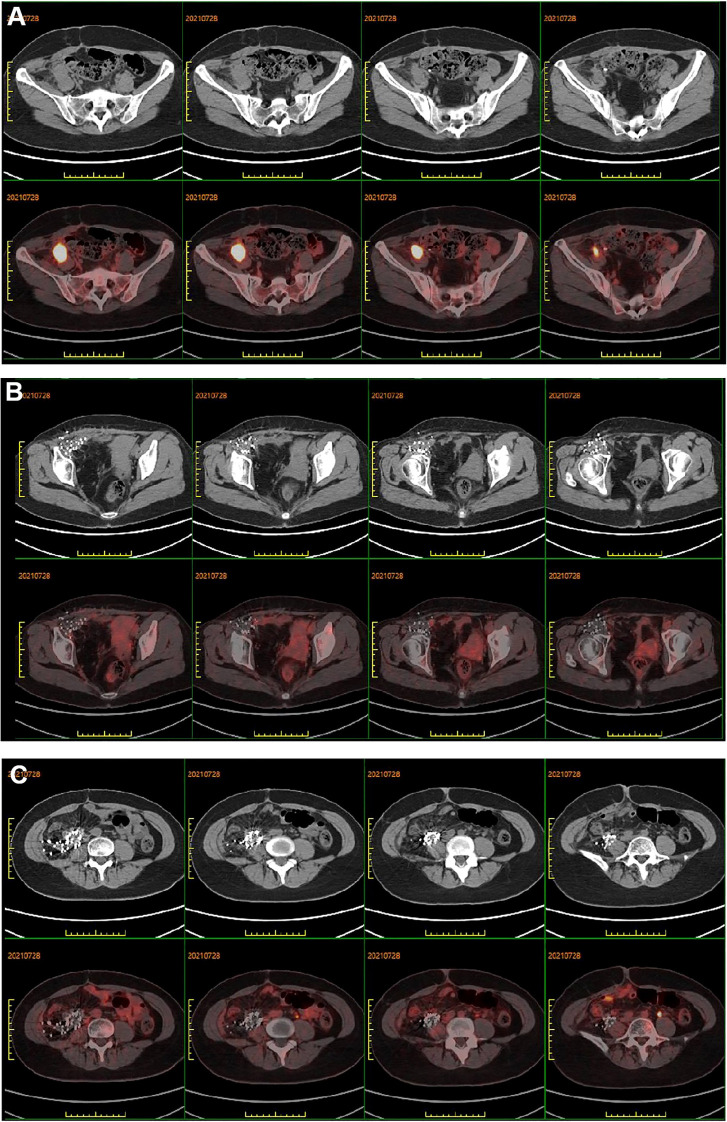
**(A)** PET-CT showed peripheral elevated FDG avidity in the right pelvic cavity. **(B, C)** PET-CT scan demonstrated no peripheral elevated FDG avidity in the right groin and right abdomen after 3 years of iodine-125 seed implantation, respectively.

**Figure 4:**
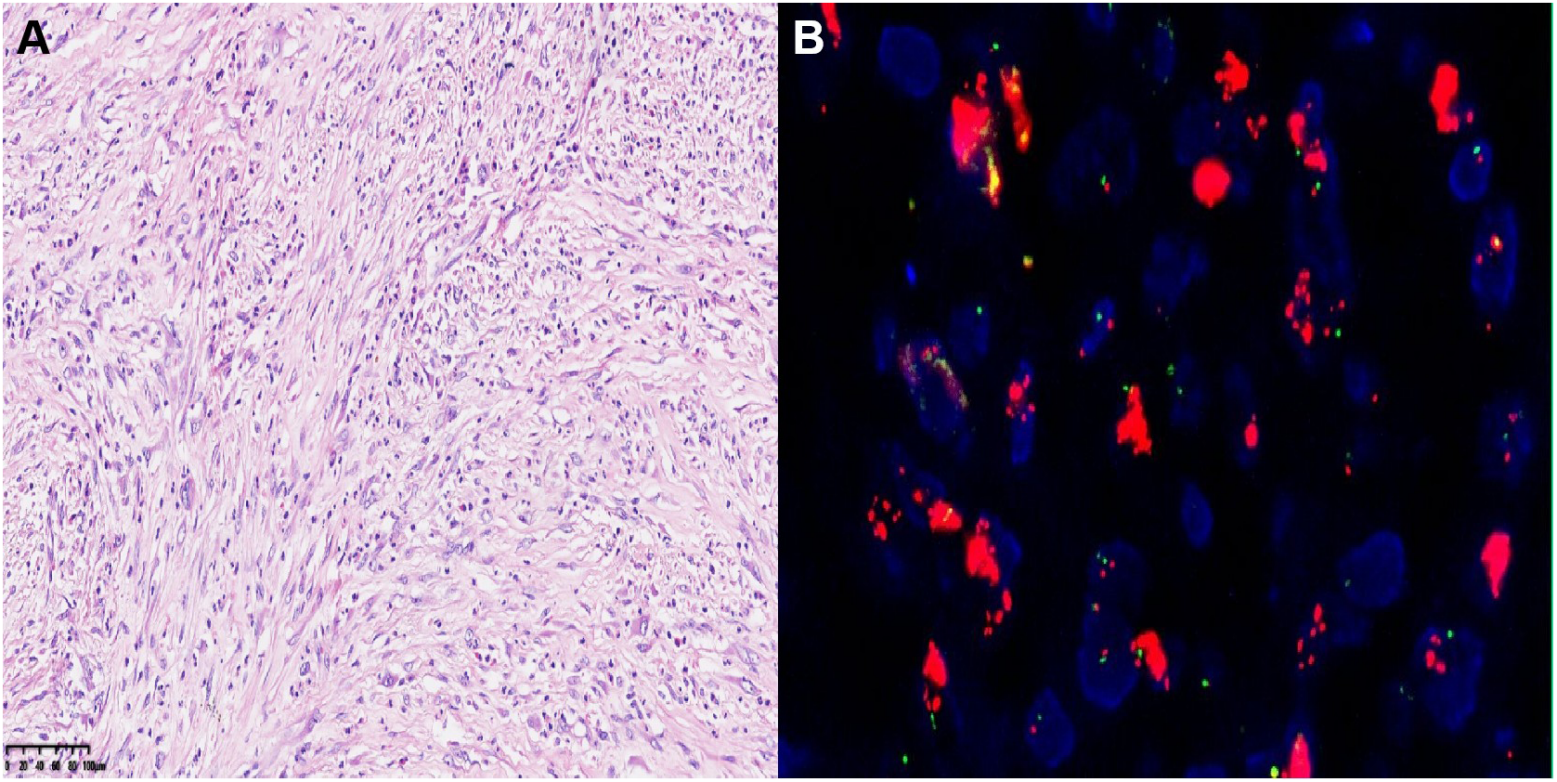
**(A)** Hematoxylin-eosin (HE) staining of postoperative tumor tissue (20×). **(B)** The FISH image showed positive result labeled by MDM2.

The patient underwent regular follow-up, and her condition remained stable (**Figures 2C-F**). However, during a CT scan in July 2021, a lesion was detected in the right pelvic region. To confirm the diagnosis, a PET-CT examination was performed, revealing a high metabolic lesion measuring 2.1 × 3.1 cm in the right pelvic region (**Figure 3A**), which was consistent with liposarcoma recurrence. Notably, the previous iodine-125 seed implantation site remained stable, and no abnormal uptake was observed on PET-CT at that location (**Figures 3B, C**). Despite the recurrence, the patient had achieved a 3-year progression-free survival (PFS) period. Subsequently, the patient underwent retroperitoneal tumor resection and partial ileocecal colon resection. Postoperative pathological examination revealed a mesenchymal tumor with significant cellular atypia, giant cells, degenerative changes, and extensive infiltration of neutrophils and eosinophils (**Figure 4A**). Immunohistochemical analysis showed the following results: Vimentin (+), S-100 (partially +), SMA (partially +), Desmin (partially +), Bcl-2 (partially +), CD34 (vascular +), ALK (-), CD117 (-), Dog-1 (-), CK (-), MyOD1 (-), and a Ki-67 index of 20%. Fluorescence *in situ* hybridization (FISH) analysis confirmed MDM2 amplification (**Figure 4B**). Based on the pathological, immunohistochemical, and genetic findings, the final diagnosis was dedifferentiated liposarcoma. Since then, the patient has been under regular follow-up, and her condition has remained stable to date (**Figures 2G, H**).

## Discussion

Liposarcoma is one of the most common subtypes of soft tissue sarcoma (STS) (9). According to the World Health Organization (WHO) classification, liposarcoma is divided into five main subtypes: dedifferentiated liposarcoma (DDLPS), myxoid liposarcoma (MLPS), pleomorphic liposarcoma (PLS), well-differentiated liposarcoma (WDLPS)/atypical lipomatous tumor, and mixed-type liposarcoma (10). These histological subtypes are closely associated with prognosis. Well-differentiated liposarcomas typically exhibit slow growth and low invasiveness, resulting in a more favorable prognosis. In contrast, dedifferentiated liposarcomas are highly invasive and metastatic, leading to a poorer prognosis (11). Currently, surgical resection remains the primary treatment for liposarcoma (12, 13). However, postoperative recurrence and metastasis rates are notably high (14). For patients who are not candidates for surgery, radiation therapy or systemic chemotherapy may be considered. Common chemotherapeutic regimens include doxorubicin alone or in combination with ifosfamide, gemcitabine alone or combined with docetaxel, or trabectedin. Unfortunately, liposarcomas generally exhibit a low response rate to these agents, and the duration of response is often short (15, 16). Recently, angiogenesis inhibitors such as anlotinib have demonstrated efficacy in the treatment of advanced and metastatic STS, including liposarcoma (17). In a phase II clinical trial, patients treated with anlotinib achieved a median progression-free survival (PFS) of 5.63 months and a median overall survival (OS) of 12.33 months (18).

In this case, the patient experienced rapid tumor recurrence shortly after undergoing extensive surgery, with the tumor being large and exhibiting aggressive growth. After a multidisciplinary team (MDT) consultation, a comprehensive assessment of the patient’s physical condition and surgical tolerance was carried out. It was concluded that complete surgical resection was challenging and was associated with a high risk of postoperative recurrence. Therefore, a second surgical intervention was considered inappropriate. Moreover, the anatomical changes resulting from the previous surgery presented significant challenges for precise localization in external beam radiation therapy. In contrast, for unresectable retroperitoneal liposarcoma, brachytherapy (internal irradiation) can effectively control tumor growth and, in some cases, even reduce the size of the tumor. Eventually, a comprehensive treatment approach combining radioactive iodine - 125 seed implantation with anlotinib targeted therapy was adopted. Iodine - 125 seeds continuously emit low - dose gamma rays with a 1.7 - cm radiation radius, effectively killing nearby cancer cells while safeguarding normal tissues. Compared to conventional external beam radiation therapy, radioactive seed implantation, as a form of internal radiation therapy, offers several advantages, including minimal invasiveness, a high radiation dose to the target area, prolonged action time, improved local control rates, and enhanced protection of surrounding normal tissues (19). This approach allows the tumor to receive a high dose of radiation while minimizing damage to adjacent healthy tissues. To further control tumor recurrence, the patient was administered oral anlotinib, a targeted therapy aimed at inhibiting tumor angiogenesis and proliferation signaling pathways. The goal of this combined treatment strategy was to achieve effective tumor control through continuous local radiation from the implanted seeds and systemic molecular targeted therapy, while minimizing treatment-related adverse effects. This approach provided a safer, more effective, and better-tolerated treatment option for the patient. Although tumor recurrence occurred three years after the initiation of particle therapy, the previously treated tumor site remained stable, demonstrating the efficacy of iodine-125 seed implantation combined with anlotinib therapy.

In summary, to the best of our knowledge, this is the first reported case demonstrating the effective antitumor activity of iodine-125 seed implantation combined with anlotinib in the treatment of liposarcoma. This combined approach highlights its potential as a promising therapeutic strategy for controlling local tumor progression and extending patient survival.

## Data Availability

The raw data supporting the conclusions of this article will be made available by the authors, without undue reservation.
